# Macrophages Sequester Clofazimine in an Intracellular Liquid Crystal-Like Supramolecular Organization

**DOI:** 10.1371/journal.pone.0047494

**Published:** 2012-10-11

**Authors:** Jason Baik, Gus R. Rosania

**Affiliations:** Department of Pharmaceutical Sciences, University of Michigan College of Pharmacy, Ann Arbor, Michigan, United States of America; University of Helsinki, Finland

## Abstract

Clofazimine is a poorly-soluble but orally-bioavailable small molecule drug that massively accumulates in macrophages when administered over prolonged periods of time. To determine whether crystal-like drug inclusions (CLDIs) that form in subcellular spaces correspond to pure clofazimine crystals, macrophages of clofazimine-fed mice were elicited with an intraperitoneal thioglycollate injection. Inside these cells, CLDIs appeared uniform in size and shape, but were sensitive to illumination. Once removed from cells, CLDIs were unstable. Unlike pure clofazimine crystals, isolated CLDIs placed in distilled water burst into small birefringent globules, which aggregated into larger clusters. Also unlike pure clofazimine crystals, CLDIs fragmented when heated, and disintegrated in alkaline media. In contrast to all other organelles, CLDIs were relatively resistant to sonication and trypsin digestion, which facilitated their biochemical isolation. The powder x-ray diffraction pattern obtained from isolated CLDIs was consistent with the diffraction pattern of liquid crystals and inconsistent with the expected molecular diffraction pattern of solid, three dimensional crystals. Observed with the transmission electron microscope (TEM), CLDIs were bounded by an atypical double-layered membrane, approximately 20 nanometers thick. CLDIs were polymorphic, but generally exhibited an internal multilayered organization, comprised of stacks of membranes 5 to 15 nanometers thick. Deep-etch, freeze-fracture electron microscopy of unfixed snap-frozen tissue samples confirmed this supramolecular organization. These results suggest that clofazimine accumulates in macrophages by forming a membrane-bound, multilayered, liquid crystal-like, semi-synthetic cytoplasmic structure.

## Introduction

Clofazimine is an antibiotic and anti-inflammatory drug that is very poorly soluble yet orally bioavailable [1], [2], [3], [4]. It is clinically approved to treat leprosy and skin inflammation associated with *Mycobacterium leprae* infection [2], [4], [5]. Clofazimine possesses three ionizable amine groups that become protonated and charged at acidic pH. It is a highly hydrophobic molecule, with a logP >7. Thus, clofazimine's solubility increases in acidic environments, but it is virtually insoluble in aqueous media at neutral or alkaline pH [6]. Therefore, in the gastrointestinal tract, clofazimine could form supersaturated solutions as it passes from the acidic pH of the stomach to the more alkaline pH of the intestine. Clofazimine possesses a large volume of distribution and its elimination half-life is more than 70 days [1], [2], [3], [4]. However, the drug's biodistribution pathways are not known. Clofazimine could bind to proteins and form complexes with intracellular membranes [6]. It could also precipitate out as particulate aggregates or crystals that may be actively phagocytosed by cells of the mononuclear phagocyte system.

Discovered in the 1950s, clofazimine is active against drug resistant strains of mycobacteria and possesses a unique spectrum of anti-inflammatory activities. It remains clinically useful to this day. However, clofazimine accumulates to very high levels in tissues [4], [7], [8], [9] resulting in visible changes in the pigmentation of skin and other organs. In patients, clofazimine has been reported to form crystal-like drug inclusions (CLDIs) in macrophages [4], [10], [11]. Clofazimine bioaccumulation is associated with various other side effects, but the drug is well-tolerated and side effects disappear upon discontinuation of treatment [10], [12], [13], [14], [15]. Because of its complex pharmacokinetics, clofazimine has been relegated to a category 5 agent.

Since the number of mycobacterial infections resistant to first line antibiotic therapy has been increasing, there is renewed interest in developing a new generation of clofazimine derivatives active against drug resistant mycobacterial strains, but with a decreased propensity to bioaccumulate. Well-informed modifications of the chemical structure of clofazimine could provide a good starting point for the development of a second generation of improved clofazimine derivatives [16]. This is particularly timely and important since phenazines are highly effective against multidrug resistant mycobacteria [17] that are responsible for drug resistant tuberculosis and leprosy epidemics which are spreading in Africa and Asia [3], [18], [19].

CLDIs found *in vivo* have been generally assumed to correspond to solid clofazimine crystals similar to the crystals that precipitate out in pure clofazimine solutions. However, CLDIs found in biological samples are too small for single crystal X-ray diffraction structural studies. Therefore, we decided to directly probe the physical and chemical properties of isolated CLDIs, using a materials science inspired approach in combination with various microscopic imaging techniques.

## Results

### 1. Long term clofazimine bioaccumulation and retention *in vivo*

**Figure 1:**
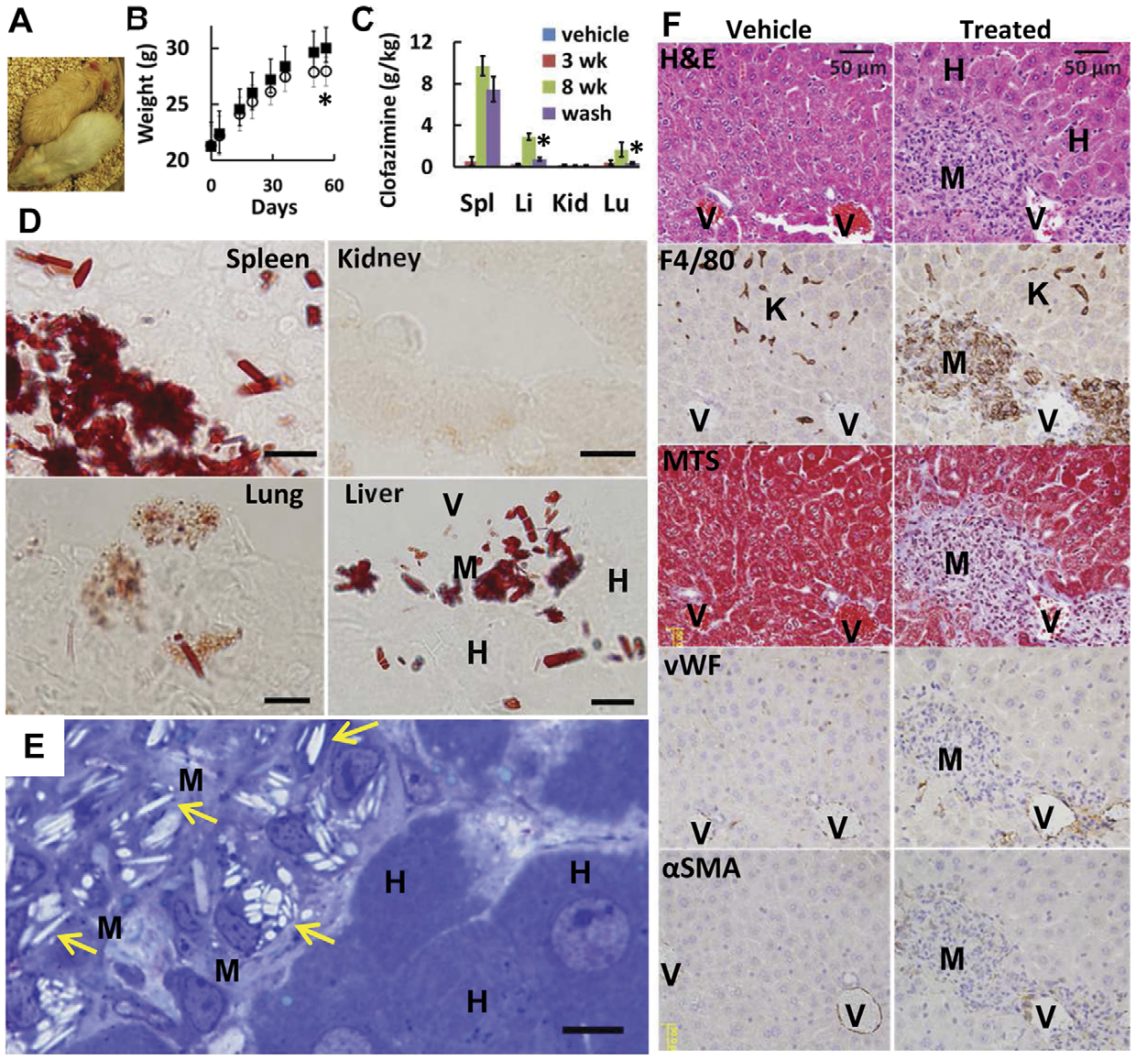
Clofazimine inclusions formed in macrophage-like cells *in vivo*. (**A**) Mice fed with clofazimine (above) showed reddish pigmentation visible in the ear, tail, and skin when compared to mice treated with vehicle only (below). (**B**) Weight gains in mice fed with and without clofazimine were comparable (N = 40, ▪, vehicle; ○, treated; *, P<0.01, end-point T-test). (**C**) Biochemical analysis of various organs revealed differences in the accumulation and retention of clofazimine after wash out (*, P<0.01, N = 5 per group, ANOVA). (**D**) Ruby red inclusions appeared in frozen sections of spleen, lung and liver, but not in kidneys of 8 wk supplemented diet. H, hepatocyte; V, blood vessel; M, microgranulomas. (**E**) Intracellular inclusions were extracted in perfusion-fixed liver upon ethanol-dehydration and staining with toluidine blue. Arrows indicate needle-like cavities remaining after extraction. (**F**) Histological sections revealed cellular changes in liver of mice fed with clofazimine. H&E staining, F4/80 macrophage specific marker, Masson's trichrome staining (MTS, collagen fibers), von Willebrand factor (vWF, endothelium) and alpha smooth muscle actin (αSMA). K, Kupffer cells. Scale bar  = 10 µm unless otherwise indicated.

For long term, continuous administration, mice were fed with powdered rodent feed supplemented with clofazimine, *ad libitum* [4], [9], [20], [21]. This resulted in an approximate daily intake of 20 mg/kg, well below clofazimine's LD50 and comparable to the human daily dose of 4.3 mg/kg [8]. As in humans [8], mice skin gradually turned red after three weeks (Fig. 1A). Otherwise, the treated animals gained weight and behaved similarly to their untreated counterparts (Fig. 1B). From 3 to 8 weeks of dietary supplementation, continuous, non-steady state accumulation occurred in spleen, liver and lung; in other organs, like the kidney, accumulation was minimal (Fig. 1C). In spleen, the mass of clofazimine almost reached 1% of the wet organ weight (Fig. 1C). After discontinuation of supplementation, mice skin gradually returned to a paler color of untreated skin, over a period of two months. However, clofazimine concentration only decreased by 76% and 73% in liver and lung, while changes in concentration in the spleen were statistically insignificant even after two months of clofazimine withdrawal. This confirmed a specific clofazimine sequestration and retention mechanism in this organ (Fig. 1C).

Consistent with previous studies [3], [20], LC/MS analysis of spleen, liver and plasma samples revealed a single drug-associated peak with a molecular weight matching clofazimine's, indicating clofazimine was present in tissues in metabolically intact form (data not shown). Examination of unfixed, unstained frozen tissue sections by transmitted light microscopy revealed dark, ruby red CLDIs present in all organs exhibiting continuous accumulation and retention of the drug (Fig. 1D). CLDIs were most numerous in the spleen and lymph nodes, followed by the liver, small intestine and lungs. In treated mice, splenomegaly (mass increase by >3.4 fold (N = 5), P<0.01; t-test) and swollen mesenteric lymph nodes were apparent [22]. At 3 week treatment, the average size of the inclusions was 2.2±0.78 (SD) µm in width and 3.9±2.6 µm in length. After continued feeding until 8 week, the individual inclusions were only slightly more elongated to 6.0±3.6 µm (P<0.01) but increased in numbers. Thus, the growth of drug inclusions appeared to be constrained by the size of the cells. After clofazimine diet was switched to a clofazimine-free, regular diet for two months to let drugs be washout out (Fig. 1C, wash), CLDIs were still present only in those organs that retained clofazimine.

### 2. CLDIs are exclusively present inside macrophage-like cells

CLDIs appeared exclusively inside macrophage-like cells (Fig. 1E), consistent with prior reports [4], [11], [20]. In liver, hepatocytes (Fig. 1E, H) clearly lacked CLDIs, whereas macrophages (Fig. 1E, M) with CLDIs formed small clusters resembling microgranulomas, which are normally formed by liver macrophages under stress [23]. This was confirmed by immunohistochemical analysis (Fig. 1F). These clusters were adjacent to blood vessels (Fig. 1F, V) and, most cells in these clusters expressed the F4/80 antigen (macrophage marker; Fig. 1F) which also labeled the Kupffer cells (Fig. 1F, K). The cell clusters were embedded in extracellular matrix (Masson's trichrome staining, MTS; Fig. 1F) and they did not stain with von Willebrand factor (endothelial cell marker, vWF; Fig. 1F) or smooth muscle actin (smooth muscle marker, αSMA; Fig. 1F).

**Figure 2:**
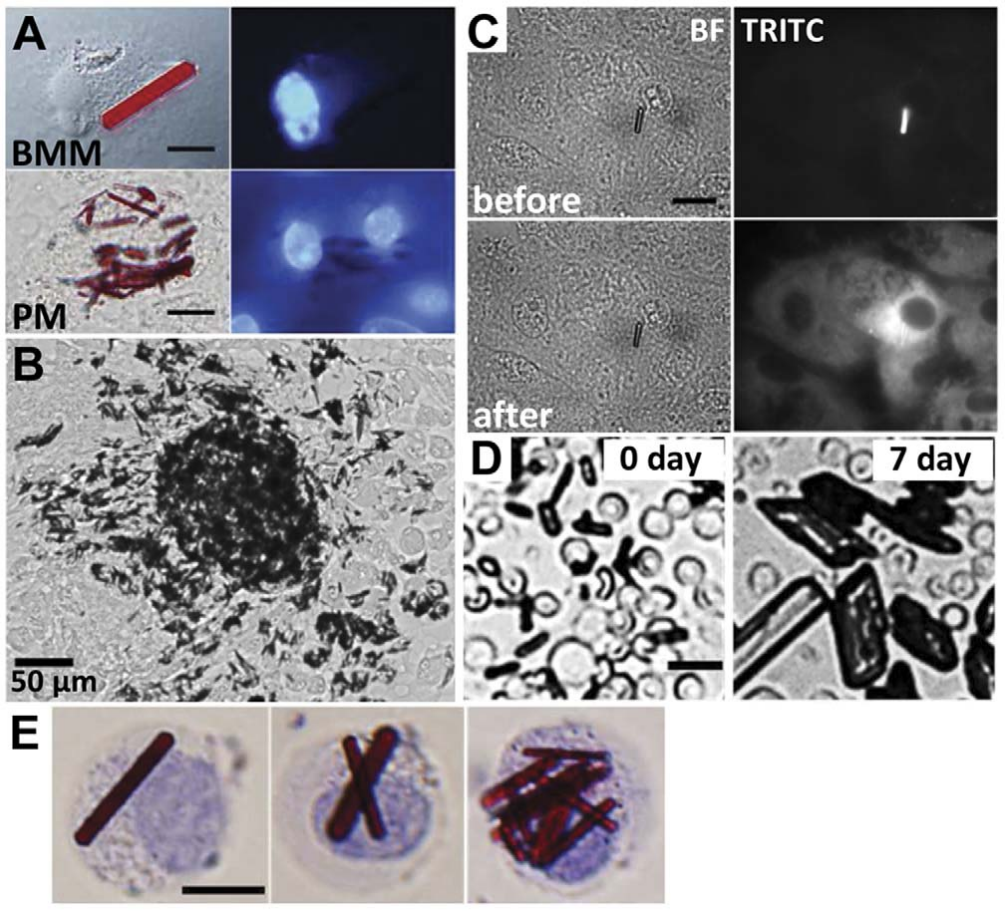
Macrophages containing intracellular CLDIs were collected, plated and studied *in vitro*. (**A**) Bone marrow macrophage (BMM) and thioglycollate elicited peritoneal macrophages (PM) were obtained from mice fed with clofazimine, attached and spread on tissue culture plastic, and were stained with Hoechst 33342 to show nuclei. (**B**) Peritoneal macrophages with CLDIs migrated away from large clusters when plated on tissue culture dishes. (**C**) Illuminating peritoneal macrophages with blue (490 nm) light triggers clofazimine release (observed in TRITC channel) from CLDIs. (**D**) Once removed from cells, extracellular CLDIs grew in size and became irregular in morphology, unlike intracellular CLDIs. Red blood cells (d = 8 µm) in the background serve as size markers, for reference. (**E**) CLDIs inside bone marrow-derived cells in suspension, stained with Trypan Blue. Scale bars  = 10 µm unless otherwise indicated.

To determine if CLDIs were associated with viable, chemotactic, adherent cells, bone marrow macrophages (BMM) were isolated from femurs and peritoneal macrophages (PM) were elicited by intraperitoneal injection of 4% thioglycollate [24] (Fig. 2). BMM and PM cells containing CLDIs were able to attach and spread on tissue culture plastic (Fig. 2A). The size and shape of the CLDIs of the elicited macrophages resembled those observed in tissue cryosections (Fig. 1). PMs containing CLDIs were viable and motile, and migrated out from clusters onto the tissue culture plastic (Fig. 2B). In these cells, CLDIs maintained their shape and size without any disintegration, degradation or dissolution. Interestingly, with peritoneal macrophages isolated *in vitro*, illuminating CLDIs with 490 nm light induced release of clofazimine which could be detected using the standard TRITC filter set of an epifluorescence microscope (Fig. 2C). Soon after illumination, clofazimine's fluorescence filled the cell. Upon continued illumination, clofazimine's fluorescence visibly diffused to the neighboring cells.

From the spleen, CLDIs could be removed from the cells by forcefully grinding the tissue homogenate and passing the homogenate through a cell strainer. Initially, CLDIs appeared morphologically homogeneous. However, CLDIs gradually transformed into irregular, rhomboidal shapes resembling typical, pure clofazimine crystals, which grew larger than cells in size (from 9.7±3.1 (SD) µm on day 0, to 15±4.7 µm on day 7; Fig. 2D). Similar results were obtained with bone marrow macrophages which exhibited varying number of intracellular CLDIs that appeared as rigid, prism-like structures after cells were rounded and detached from the plastic (Fig. 2E).

### 3. CLDIs possess physicochemical properties different from those of pure clofazimine crystals

**Figure 3:**
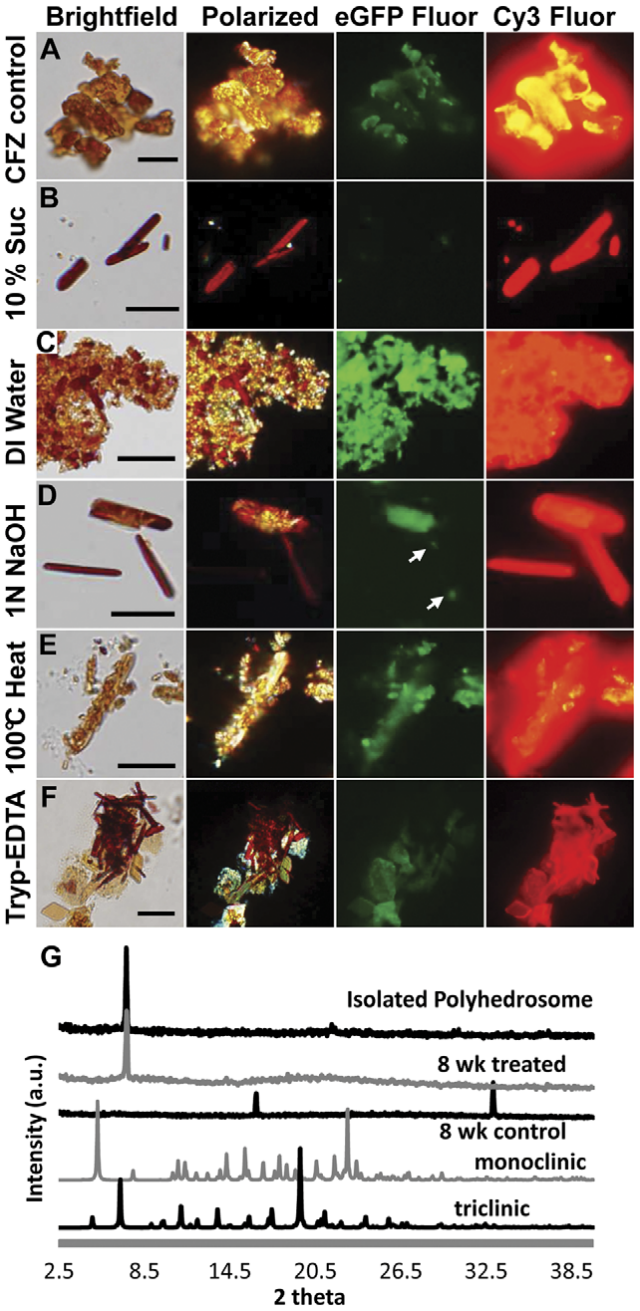
CLDIs exhibited different chemical and physical properties from pure clofazimine crystals. Polarized light and epifluorescence microscopy (using the eGFP or Cy3 fluorescence channels) showed that pure clofazimine crystals (control, **A**) were unchanged by different treatments. These crystals appeared birefringent and fluoresced in the standard eGFP and Cy3 channels of the epifluorescence microscope. (**B**) Isolated CLDIs remained intact in isotonic solution of 10% sucrose in water, did not fluoresce in the eGFP channel but fluoresced in the Cy3 channel. (**C**) Isolated CLDIs burst and aggregated in distilled water, and became fluorescent in the eGFP channel. (**D**) After exposure to 1N NaOH, isolated CLDIs partially disintegrated in different parts. Arrows point to the tips of a CLDI that were fluorescent in the eGFP channel. (**E**) After 15 min at 100°C, CLDIs fragmented and changed to a pale orange color. (**F**) CLDIs appeared to remain partly intact when viewed after 30 min sonication and 1 hour trypsin treatment. Scale bars  = 10 µm. (**G**) Powder X-ray diffractogram for isolated CLDI and 8 wk treated mouse spleen homogenate showed a single peak at 2-theta  = 7.2°Control spleen homogenate from vehicle-only treated mouse did not show this peak. As a reference, pure, solid clofazimine crystals (monoclinic and triclinic) showed many peaks at higher angle indicative of a three dimensional, molecular lattice organization.

We proceeded to compare some of the physical and chemical properties of isolated CLDIs directly with those of pure clofazimine crystals [7], [10], [14], [15], [25]. Clofazimine crystals were irregular in shape and size (Fig. 3A). The pure clofazimine crystal powder obtained from the manufacturer fluoresced in the standard, green (eGFP) channel and in the red (Cy3) channel of the epifluorescence microscope. Their fluorescence or morphology did not change when they were placed in water and heated to 100°C, placed in 1N NaOH or trypsinized (the melting point of pure clofazimine crystals is 212°C).

For comparison, CLDIs were isolated by mincing and disaggregating spleens with 0.125% trypsin-EDTA, ultrasonicating them for 30 minutes after passing them through a cell strainer. CLDIs were the only microscopic structure visibly remaining in the filtrates, and could be concentrated by centrifugation. Isolated CLDIs in the pellets could be resuspended in 10% sucrose in water, and appeared stable when examined within a couple of hours after isolation. They were dark, ruby red in color, and prism-like in appearance when viewed with transmitted light. They were birefringent when viewed with polarized light. Unlike pure clofazimine crystals, the isolated CLDIs were homogenous in shape and size, and polarized light as a single domain. They appeared bright red and monolithic when viewed using cross-polarizers, in contrast to the heterogeneous, yellow-orange fragmented birefringence of pure clofazimine crystals. Unlike pure clofazimine crystals, isolated CLDIs did not fluoresce in the green eGFP channel, yet they were brightly fluorescent in the red Cy3 channel (Fig. 3B).

Unlike clofazimine crystals, isolated CLDIs were highly responsive to changes in the environment. Upon exposure to distilled water (Fig. 3C), they burst into smaller birefringent globules which aggregated into large masses and became fluorescent in the green eGFP channel. Upon treatment with 1N NaOH (Fig. 3D), they underwent localized changes in structure: parts of the elongated CLDIs fragmented and transformed to the green eGFP fluorescent form. Other CLDIs treated in this manner became fluorescent at the tips (arrows). Isolated CLDIs in suspension disintegrated when heated to 100°C for 15 minutes (Fig. 3E). When heated, they turned yellow, became fluorescent in the green eGFP channel, burst into small globules that remained attached without aggregating to each other. Morphologically, CLDIs appeared relatively resistant to 0.125% trypsin-EDTA treatment for 1 hour (Fig. 3F).

To determine whether CLDIs possessed subnanometer molecular features associated with the three-dimensional lattice structure of solid clofazimine crystals, powder X-ray diffraction analysis was performed on isolated CLDI samples. Based on the powder X-ray diffraction pattern of isolated CLDIs (Fig. 3G), a single peak was observed at a small diffraction angle. The absence of other diffraction peaks at higher angles was noteworthy, as those peaks correspond to the subnanometer features of the three dimensional lattice structure of pure, solid clofazimine crystals [26].

### 4. Electron microscopy reveals the internal organization of CLDIs

**Figure 4:**
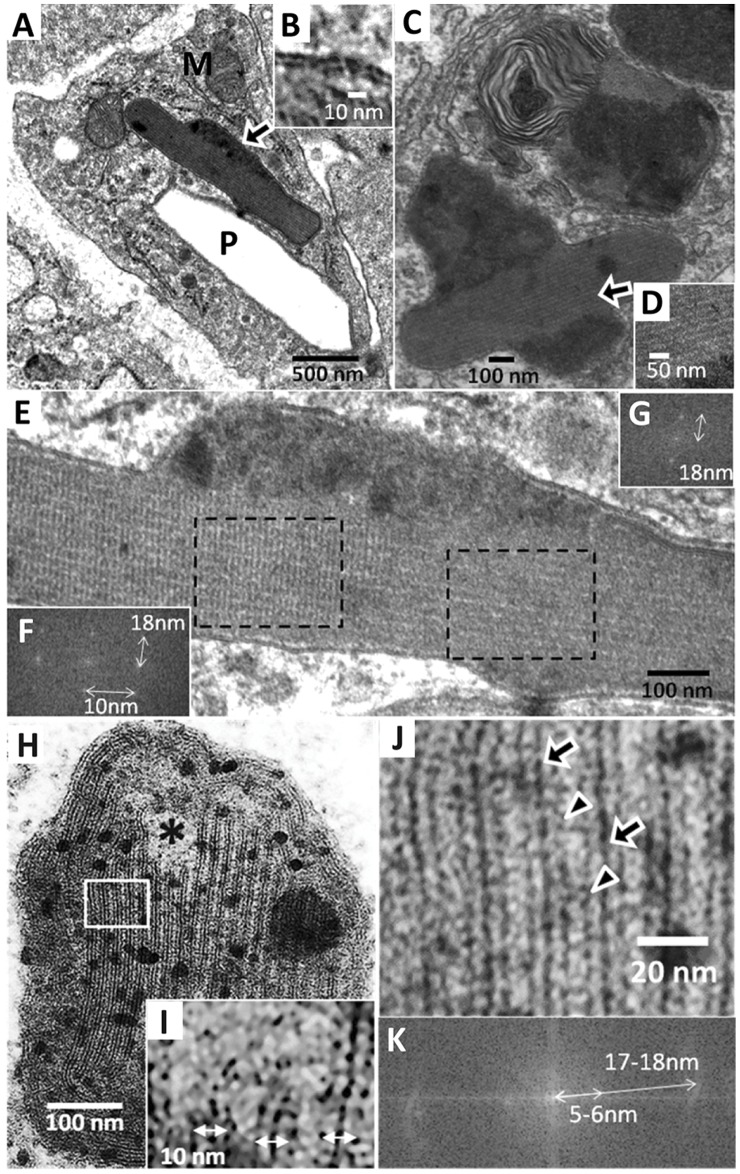
TEM and deep-etch freeze fracture electron microscopy analysis of CLDIs. (**A**) Intact CLDIs were observed in the cells of the lamina propria of 8.5 wk treated mouse jejunum using TEM. P, extracted polyhedral cavities. M, mitochondria. (**B**) CLDIs were delimited by a lipid double layer. (**C**) CLDIs appeared to form from heterogeneous granular domains transforming into a lamellar structure, observed in 4.5 wk treated jejunum (**D**). (**E**) Zoomed image of the CLDI from (A) showed the lattice-like lamellar structure. (**F**) and (**G**), Discrete Fourier Transforms confirmed the regular, periodic structure of CLDIs. (**H**) Transversal cross section of CLDIs from 8.5 wk jejunum showed an internal organization of parallel bands and some amorphous regions indicated by (*). (**I**) Zoomed image revealed the trilayer membrane of 10 nm in width separated by inter-laminar space continuous with amorphous region. (**J**) Zoomed image of rectangle in (H), showing trilayer membrane structure comprised of a central dark band (arrows) flanked by a pair of less prominent, dark bands (triangles) on either side, separated by clear 5 nm space. The trilayer membranes were regularly spaced and formed planar stacks, with the central bands exhibiting the 18 nm spacing in the Discrete Fourier Transform (**K**).

In transmission electron microscope images, CLDIs generally appeared as empty, featureless polyhedral cavities. Superficially, the outline of these cavities resembled the faceted outline of pure crystals, as has been previously reported [4], [11]. Nevertheless, some CLDIs observed in our samples were filled with osmiophilic material (Fig. 4A). These osmiophilic bodies of filled CLDIs were also elongated and polyhedral in shape, and bounded by a double membrane (Fig. 4B). In some cells, other atypical transitional organelles were observed. These putative, transitional organelles appeared as heterogeneous granular or multivesicular bodies deformed by an internal, elongated CLDI-like structure, or multilamellar bodies that appeared to be in the process of transforming or fusing with granular or multivesicular bodies (Fig. 4C).

At higher magnification, visual inspection of these CLDIs revealed morphological details in the 1 to 20 nanometer scale. As seen in medial cross-sections cut along the long axis of the object (Fig. 4D and 4E), the filled CLDI contained a multilamellar core comprised of planar sheets separated by parallel array of clear “channels”. These channels were spaced 18 nm apart, and aligned parallel to the long axis of the structure (Fig. 4F and 4G). In other regions, the CLDI appeared as a lattice with periodically-spaced elements repeating every 10 or 18 nm (Fig. 4F).

Transversal cross-sections cut perpendicularly to the long axis (Fig. 4H) revealed a multilamellar core comprised of planar stack of trilayer membranes of 10 nm thickness (Fig. 4I). The entire structure was bounded by an outermost double membrane, about 20 nm thick (Fig. 4H). The core was surrounded by concentric lamellae of cortical, trilayer membranes which were observed in all the CLDIs and the transitional structures (Fig. 4C). The trilayer membrane consisted of a thick, dark osmiophilic band at the center (Fig. 4J, arrows) flanked by two thinner osmiophilic bands on either side (Fig. 4J, triangles). A clear, 5 nm layer separated the central band and the flanking bands (Fig. 4K). These trilayer membranes sometimes merged with membrane-free regions (Fig. 4H, *).

### 5. Deep-etch freeze-fracture electron microscopy on intact CLDIs

**Figure 5:**
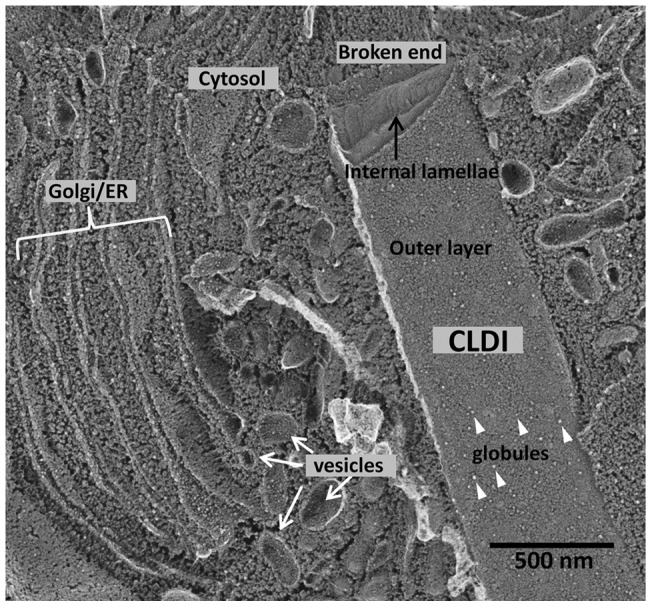
Deep-etch freeze-fracture microscopy of the intracellular CLDI from 6 wk treated mouse liver. Note the outer membrane of the CLDI is studded with globular protein-like features (white triangle). The CLDI was broken during freeze-fracture, revealing a lamellar internal structure at the top.

Remarkably, this intracellular multilayered structure appeared different from all other multilamellar organelles previously reported inside cells [27]. Thus, we also considered the possibility that the observed morphological features could be an artifact of the transmission electron microscopy sample preparation technique. To study the morphology of CLDIs as closely to their native state as possible, we turned to a completely different sample preparation technique: deep-etch freeze-fracture electron microscopy [28]. This technique creates a platinum replica of a snap-frozen tissue sample after surface layers of frozen water molecules are sublimated in vacuum. By eliminating the fixation, dehydration and polymerization steps used in transmitted electron microscopy, the deep-etch freeze-fracture technique preserves the topography of cellular membranes with high fidelity. Remarkably, in the sample of unfixed liver of 6 wk treated mouse, CLDIs appeared prominently and clearly stood out from the rest of the cytoplasm (Fig. 5). Their size, shape, location and general morphology was consistent with the results obtained with TEM studies. Viewed from the outside, CLDIs appeared surrounded by cellular membrane with protein-like structural features similar to those present in the other cellular membranes in the cytoplasm of the cell (white triangle, Fig. 5).

**Figure 6:**
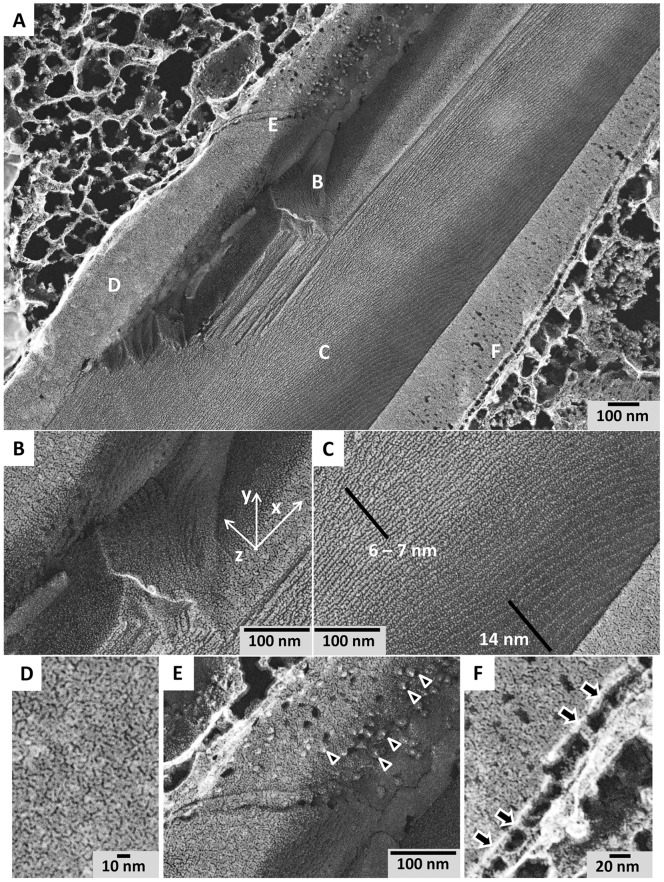
Outer membrane and internal organization of a CLDI from 6 wk treated mouse liver. (**A**) Zoomed out image, revealing the exposed, inner multilamellar core with the outer double membrane layers peeled back towards the cytoplasm of the cell. The CLDI was broken open during the sample preparation process. Regions of interest (corresponding to panels B to F) are marked with letters. (**B**) Zoomed image of the inner lamellar surface. (**C**) Zoomed image of the multilamellar core region, showing variable spacings in the order of ranging from 6 to 14 nanometers. (**D**) Zoomed image of the innermost face of the inner bounding double membrane without the globular, protein-like features. (**E**) Zoomed image of the innermost face of the outer bounding double membrane. Protein-sized features are observed on the inner surface of the outer membrane (arrowheads). (**F**) Zoomed image of the outer double membrane. Note the large space between the double membranes, with “pillars” bridging the membranes (arrows).

Inside the CLDI, deep-etch freeze-fracture EM images revealed evidence of a multilayered structure at the core surrounded by a double membrane (Fig. 6A). Zooming into the core (Fig. 6B), the multilayered structure appeared stacked in the z direction, perpendicular to the long axis of the CLDI. No lateral organization was visible in the xy plane. The lamellar spacing and orientation of the layered planes was not always regular due to uneven fracture, and ranged from 6 nm to 14 nm at different points inside the structure (Fig. 6C). Consistent with a liquid crystal-like structure, there was no obvious lateral organization along the planar surface of each lamellae (Fig. 6D). We also noted that the core of the structure did not seem to possess protein-like globular features (Fig. 6D). Protein-like globular features were only observed in the outer or inner face of the outer membrane covering the entire structure (Fig. 6E, arrowheads). As in TEM images (Fig. 4B) the entire structure was surrounded by a double membrane (Fig. 6F). However the intermembrane space was in the range of 20 to 30 nanometers thick, with “pillars” bridging the outer and inner membrane (Fig. 6F, arrows).

**Figure 7:**
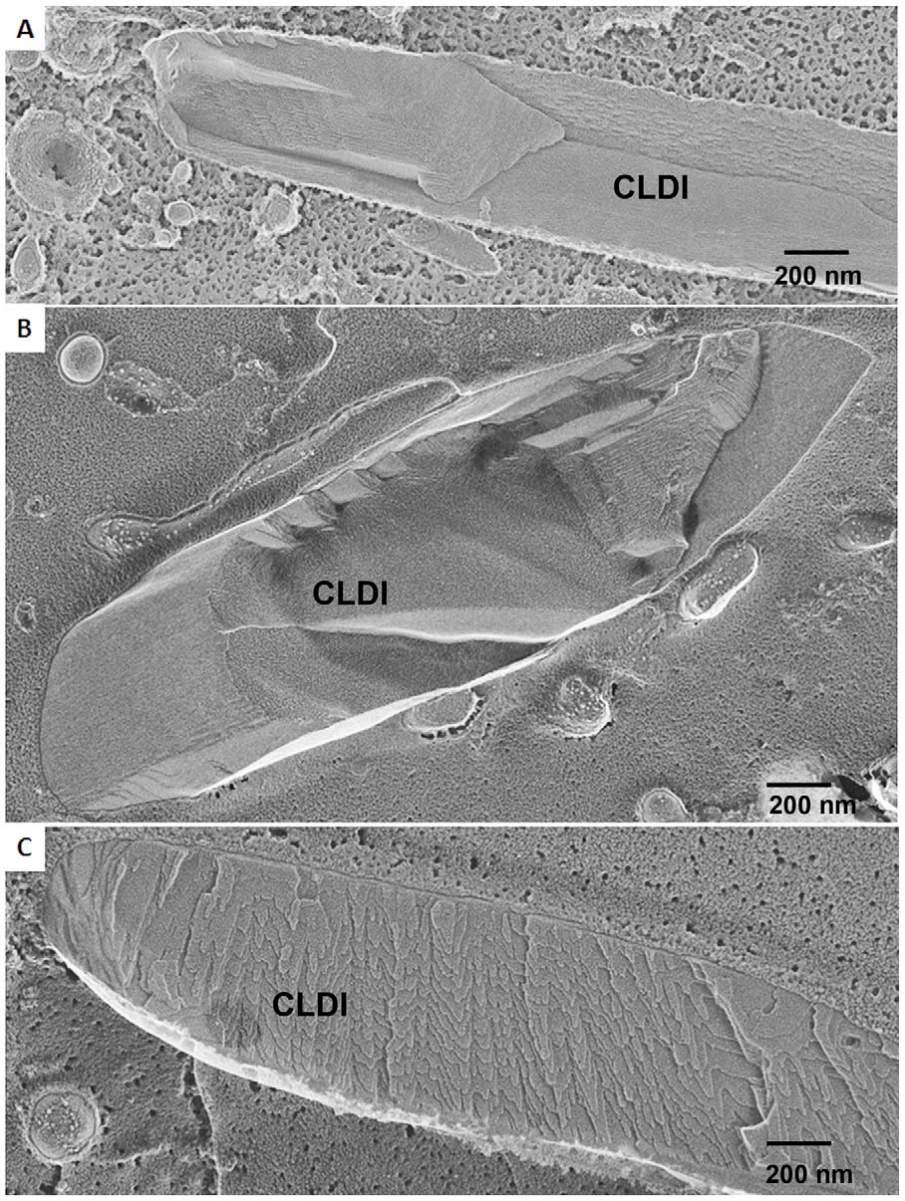
Deep-etch freeze-fracture electron microscopy of isolated CLDIs. (**A**) Pure isolated CLDIs stood out from surrounding ice and cytosolic debris based on their elongated polyhedral shape and internal layered structure. (**B**) Isolated CLDIs clearly lacked the outer double membrane covering. (**C**) Biochemically-isolated CLDI often showed outer layers of material that appeared to be peeling off from the structure.

To confirm that the structures observed in tissue samples corresponded to the biochemically isolated, purified CLDIs, we performed deep-etch freeze-fracture electron microscopy on isolated CLDI preparations (Fig. 7). In these preparations, CLDIs were readily apparent based on their shape and multilayered internal organization from cytosolic debris in the background (Fig. 7A). Nevertheless, unlike CLDIs observed in tissue samples (Fig. 5, 6), isolated CLDIs lacked the outer double membrane (Fig. 7B, C). We also noticed that, unlike CLDIs from intact tissues (Fig. 6), the internal layers of isolated CLDIs appeared to be peeling off (Fig. 7C), suggesting a partial disassembly.

## Discussion

This is the first study to directly probe the physical and chemical properties of CLDIs, and to directly reveal the morphology of CLDIs formed in clofazimine-treated animals. Initially, we considered the possibility that clofazimine accumulation in macrophages would result from the phagocytosis of extracellular clofazimine crystals by macrophages. However, extracellular clofazimine crystals were not observed *in vivo*. Chemically and physically, the intracellular CLDIs appeared very different from chemically-pure clofazimine crystals: they were uniform in size and shape, and were highly responsive to changes in medium tonicity, pH, temperature and illumination. While CLDIs have different birefringence pattern from pure crystals, this is a general property shared by many different kinds of anisotropic supramolecular organizations. Based on their stimulus-responsiveness, morphological characteristics and powder X-ray diffraction pattern, CLDIs do not resemble the three dimensional molecular arrangement of clofazimine molecules present in pure, solid, clofazimine crystals [26]. CLDIs are best described as a new kind of semi-synthetic biomaterial: a membrane-bound, stimulus responsive, macrophage-dependent, liquid crystal-like, supramolecular organization.

Interestingly, the intracellular location and growth of CLDIs appears to be exclusively constrained to the cytoplasm of macrophages. In the liver, CLDIs never appeared in association with hepatocytes. Accordingly, we hypothesize that the cell type-specific localization of CLDIs likely reflects the presence of an active intracellular sequestration mechanism that is present in macrophages and absent in other cells of the host. The presence of CLDIs in thioglycollate-elicited macrophages confirmed that these structures are found inside viable, functioning, chemotactic cells. Once removed from these cells, CLDIs were unstable: they transformed to irregular shapes and began to grow in size like a typical clofazimine crystal. Nevertheless, inside the organism, CLDIs remained uniform in shape and smaller than cells in size. This suggests an active role of the macrophages in terms of controlling the size and shape of the CLDIs.

CLDIs appeared different from all the other cellular organelles. Mechanically, their core structure was rigid and resisted sonication. In addition, three different lines of evidence suggest that their internal organization corresponded to that of a supramolecular liquid crystal: 1) transmission electron microscopy of fixed and stained samples revealed and highly organized multilamellar structure; 2) powder X-ray diffraction patterns revealed a single low angle diffraction peak consistent with a planar organization in a single spatial dimensions and no evidence of higher angle diffraction peaks that would be consistent with a three dimensional, pure crystal; 3) deep-etch freeze-fracture microscopy revealed the presence of a 2D, layered structure, with no evidence of lateral organization along the plane of each layer. The thickness of the layers observed by TEM and freeze-fracture microscopy were in the order of 5 to 15 nanometers, which is too large for the expected subnanometer features of a pure clofazimine crystal [26]. Interestingly, in deep-etch freeze-fracture preparations, there were no indications of protein-sized globular features present inside the CLDIs. Instead, the scale of supramolecular features observed by freeze-fracture microscopy, together with their response to changes in temperature, osmolarity and pH is most analogous to the supamolecular structure and phase transition behaviors of liquid crystalline mesophases adopted by concentrated phospholipids in aqueous media [29], [30], [31], [32], [33].

Based on the absence of acute toxicity *in vivo*, we propose that the sequestration of clofazimine in CLDIs may primarily serve as a defense mechanism. By sequestering clofazimine, CLDIs may have a net cytoprotective effect, reducing the concentration of soluble clofazimine molecules that would be toxic to the host. Like clofazimine [6], other compounds that induce the formation of autophagosome-like membrane complexes have also been found to possess beneficial, cytoprotective effects [6], [39], [40]. When assayed *in vitro*, clofazimine disrupts mitochondrial membrane potential and inhibits the growth of cells in tissue culture [6], [34]. In solution, clofazimine can generate superoxide anions upon interaction with isolated rat peritoneal macrophages [35] and human neutrophils [36] which may be related to its *in vitro* cytotoxic activity [34], [37]. Superoxide production has been proposed to account for clofazimine's broad bactericidal activity against many different microorganisms including *Mycobacterium tuberculosis* [38], *Staphylococcus aureus*, and *Escherichia coli* [37]. However, when mice were treated with clofazimine, there were no obvious toxicological manifestations. In humans, clofazimine is well tolerated, with gastrointestinal disorders being the major toxicological side effect manifested after long term treatment [10], [15], [25]. Nevertheless, this side effect is reversible and subsides after treatment is discontinued.

In relation to other chemotherapeutic agents in clinical use, clofazimine has many unique pharmacokinetic properties. In humans, clofazimine exhibits a very long half-life. Because of its high logP, clofazimine would be expected to be distributed mostly in association with body fat. Thus, the local accumulation of clofazimine in tissue macrophages most likely reflects an active transport mechanism that promotes the influx or retention of clofazimine in these cells. Because macrophages are actively involved in the body's defense against bacterial infection, the accumulation of clofazimine in macrophages could effectively serve to mobilize clofazimine to its site of action. Therefore, although excessive bioaccumulation in macrophages may be related to some of the drug's undesirable side effects, the accumulation of clofazimine in macrophages could be therapeutically advantageous. This observation has important implications for the design of macrophage-targeted chemotherapeutic agents.

To the extent that CLDIs massively sequester clofazimine inside macrophages, our results also suggest a potential role of the immune system as a determinant of drug distribution and disposition. The specific accumulation of clofazimine in some, but not all macrophages suggests there may be a specialized subpopulation of macrophages involved in xenobiotic sequestration. Interestingly, clofazimine possesses potent anti-inflammatory activity in the clinic which makes it especially useful in the treatment of erythema nodosum, a skin inflammation that accompanies *M. leprae* infection. Thus, clofazimine's bioaccumulation in macrophages may also be associated with downstream immunomodulatory activity. By monitoring changes in immune system-related signaling molecules, it should be possible to determine whether bioaccumulation of clofazimine in macrophages activates a natural anti-inflammatory pathway that may serve to protect the host from bioaccumulation-related injury.

Previously, many *in vitro* QSAR studies have been published exploring the relationship between the chemical structure of clofazimine and its antimycobacterial properties. Most of these studies have focused on assaying the properties of phenazine molecules in solution: For example, probing how the redox properties of clofazimine depend on the type of alkylimino group at position 2 of the phenazine ring structure [34], [41]. Only recently, structure-activity relationship studies have been aimed at identifying phenazine compounds that inhibit the growth of *Mycobacterium tuberculosis* while possessing reduced potential for bioaccumulation [16]. Interestingly, the lipophilicity of clofazimine derivatives (clogP) [16] does not appear to correlate with their serum half-life, suggesting that topological features may be as important as physicochemical properties in terms of determining clofazimine's bioaccumulation and biodistribution. Using intracellular crystal formation as an endpoint, we are currently performing QSAR studies to elucidate how the physicochemical and topological features of clofazimine impact its cellular pharmacokinetics. By screening these compounds for activity against *M. tuberculosis*, these QSAR studies should facilitate the design of new phenazine derivatives with different tissue distribution and bioaccumulation potential, and may help identify an improved drug candidate with increased efficacy against *M. tuberculosis*.

To conclude, the results presented in this study constitute evidence that macrophages sequester clofazimine by forming a complex, multilayered supramolecular organization. This organization bears many unique structural features that are unlike those of natural organelles of eukaryotic cells and unlike those of chemically-pure clofazimine crystals. The distinctive physical, chemical and biological properties of CLDIs set them in a class of their own. Based on the presence of organelles with transitional morphologies (Fig. 4C), we propose CLDIs may be derived from multilamellar drug-membrane aggregates that have been observed to form in the presence of clofazimine and other drugs [6], [42]. More direct insights into CLDI structure may be possible in the near future, with higher resolution, single particle microdiffraction studies. Because CLDI formation may be an important mechanistic determinant of both clofazimine's efficacy and toxicity properties, ongoing and future studies will aim to establish the extent to which different topological features and physicochemical properties of clofazimine and related phenazine compounds lead to intracellular CLDI formation. Understanding the upstream and downstream effects of macrophages on clofazimine bioaccumulation and distribution, and the role of CLDI formation on clofazimine's pharmaco- and toxico-kinetics, should facilitate development of next generation clofazimine derivatives against multidrug resistant mycobacterial infections.

## Materials and Methods

### Ethics statement

All animal studies and procedures were performed as approved by University of Michigan Committee of Use and Care of Animals.

### Dosing protocol and TEM imaging

Mice (4–5 wk male Balb/c, Jackson Labs, Maine) were fed with drug with powder feed (3 mg/ml clofazimine (Sigma, C8895) in sesame oil, mixed at 0.01% oil to feed). Blood was collected from euthanized mice and fixed by perfusing 0.1M Sorensen's buffer and Karnovsky's fixative (3% paraformaldehyde, 2.5% glutaraldehyde) infused to left ventricle and egressed to vena cava (2.5 ml/min). Tissues were minced smaller than 1 mm in each dimension followed by TEM sample preparation and imaging, as previously described [6]. Control mice were fed with 0.01% oil to feed, and wash out mice were fed drug- and oil-free diet.

### Biochemical analysis of clofazimine in tissues

At predetermined time points, mice were euthanized using CO_2_, and blood was removed through cardiac puncture. Next, the organs were collected, washed in cold DPBS, and kept at −20°C until further analysis. Tissue (0.05–0.1 g/ml water) was homogenized with Tissumizer (Tekmar®, Cincinnati, OH), extracted with dichloromethane twice and the solvent was evaporated [9]. For measurement, clofazimine was dissolved again in methanol and its absorbance was measured at 490 nm. Concentration was calculated using a standard curve generated by spiking extracted tissue of the control (vehicle-only treated) mice tissue with known amounts of drug. Extraction yield was 60–80%.

### Immunohistochemistry

Tissues were perfusion fixed as in TEM imaging, paraffin-embedded and stained with the standard H&E and Masson's trichrome technique. Horse Radish Peroxidase and intelliPATH FLX DAB chromogen kit (IPK5010, Biocare Medical, Concord, CA) was used for anti-F4/80 (1/100, ab6640, abcam®), α-SMA (1/200, ab5694, abcam®), and vWF (1/500, ab7356, Millipore) antibody staining. All staining was performed by the Pathology Core for Animal Research (PCAR) in the Unit for Laboratory Animal Medicine (ULAM) at the University of Michigan.

### Primary cell isolation and culture

4% Brewer's thioglycollate medium was prepared sterile as described [24]. A 2 ml volume of solution was injected IP. Cells were collected 4 days later using cold DPBS. Bone marrow macrophages were flushed out from mice femurs using a fine needle syringe with DPBS [43]. Cells from spleen and lymph nodes were collected by mincing small tissue using a cell strainer with 100 µm mesh size. Cells were seeded in tissue culture plates and kept 7 days in 37°C, 5% CO_2_ with DMEM supplemented with 10% FBS, Pen/Strep, and non-essential amino acids.

### CLDI isolation

Tissue homogenate was sonicated for 30 minutes, centrifuged (100 g ×1 min) to remove large cell debris. Supernatant was resuspended in 0.125% Trypsin-EDTA solution (Gibco) and kept at 37°C for 1 hour, followed by centrifugation at 100 g to remove large cell debris. The drug inclusions in supernatant were then pelleted by centrifugation (21,000 g ×1 min), and resuspended in water. Protein content was determined with the BCA assay (Pierce 23227, Thermo Scientific) and clofazimine content was determined spectrophotometrically. For protein assay, equal volume of 5% SDS solution was mixed with the samples and the protein content was measured following the BCA kit instructions. The calculated clofazimine content normalized to the protein content indicated that the isolation procedure enriched as much as 16-fold for 8 wk treated spleen homogenate. Greater than 90% of the total clofazimine mass in the homogenates was recovered in the CLDI fraction while 95% of proteins were removed.

### Powder X-ray diffraction of clofazimine crystals and isolated CLDIs

PXRD of dried samples of isolated CLDIs and 8 wk treated (or control) mouse tissue homogenate were carried out with benchtop Rigaku Miniflex X-ray diffractometer (Danvers, MA). CuKα radiation (λ = 1.54Å), tube voltage  = 30 kV, tube current  = 15 mA. Data were collected at 2 theta from 2.5 to 40 at a continuous scan at the rate of 2.5°/min. Diffractograms of triclinic and monoclinic form of clofazimine crystals were imported from Cambridge Structural Database (CSD) and positive control of clofazimine crystal from the bottle was used for comparisons.

### Deep-etch, freeze-fracture EM

Unfixed liver was collected after exsanguination and processed for freeze-etch EM analysis, as reported previously [28] with minor modifications. In brief, all samples were kept at 4°C after removal from the animals. Samples were quickly frozen against a copper block, cooled with liquid helium using slam freezing and kept in liquid nitrogen. The sample was fractured with Balzers 400 nitrogen cooled vacuum evaporator and freeze-etched for 2 min at −100°C. Rotary replica was generated with 2 nm platinum and backed with 10 nm carbon film support. It was cleaned with chromo-sulfuric cleaning solution (Fisher Scientific, cat# SC88) for 12 hours and rinsed with DI water. The sample was picked up on formvar coated grids for viewing on a JEOL 1400 electron microscope with AMT camera.
